# Screening of endothelial dysfunction in rheumatoid arthritis in Black African subjects in the Physiology Department of the Faculty of Medicine of Dakar: a cross-sectional study

**DOI:** 10.11604/pamj.2025.50.75.40822

**Published:** 2025-05-14

**Authors:** Maïmouna Touré, Ibrahima Diouf, Fatou Bintou Sar, Coumba Diouf, Modou Oumy Kane, Abdoulaye Ba, Lamine Gueye, Abdoulaye Samb, Mamadou Sarr

**Affiliations:** 1 Laboratory of Physiology and Functional Explorations, Faculty of Medicine, Pharmacy and Dentistry, UCAD, BP 5005, Dakar Fann, Senegal,; 2 IRL3189, Environment, Health, Societies, CNRS, CNRST, Bamoko-UCAD University, Dakar, Senegal,; 3 Laboratory of Physiology Pharmaceutical/Faculty of Medicine, Pharmacy and Dentistry, UCAD, Dakar, Senegal,; 4 Laboratory of Physiology, Faculty of Health Sciences, Thies, Senegal,; 5 Rheumatology Department *CHU* LeDantec, UCAD, Dakar, Senegal

**Keywords:** Reactive hyperemia index, endothelial dysfunction, rheumatoid arthritis, Black African

## Abstract

**Introduction:**

the vascular endothelium plays an essential role in many basic physiological vascular regulations. Endothelial dysfunction is the first stage of atherosclerosis. The latter would be the main basis of the cardiovascular events responsible for the high mortality of rheumatoid arthritis. The aim of this study was to screen for subclinical endothelial dysfunction in rheumatoid arthritis in a quick, easy and non-operator dependent way.

**Methods:**

this is a cross-sectional and prospective study conducted in the department of physiology and functional explorations from the Faculty of Medicine, of Pharmacy and of Odontostomatology (FMPOS) of the Cheikh Anta Diop University (UCAD) of Dakar in Senegal. Were excluded patients under 18 years and those with rheumatoid arthritis was associated with severe complications or other medical conditions or pregnancy. Therefore, we included subjects, aged 18 and over, with rheumatoid arthritis without clinically detectable cardiovascular complications. The reactive hyperemia index (RHI) was measured by an EndoPAT2000® (Itamar Medical Ltd-Israel) to explore endothelial function. Biochemical parameters were analyzed from fasting serum and plasma using an automated Abbott device. We used SPSS software to study endothelial dysfunction and the factors associated with it for simple and multiple linear regression calculations.

**Results:**

the mean age of the study population was 42.84 ± 12.80 years. The sex ratio (M/F) was 0.19. The reactive hyperemic index was significantly lower than the normal value in more than half of the patients with rheumatoid arthritis (11 subjects out of the 19 patients, i.e. 57.89%), indicating endothelial dysfunction in these patients. After Spearman correlation tests, a positive correlation was found between reactive hyperemic index and systolic blood pressure (rho=0.753 p=0.0002), mean blood pressure (rho= 0.660 p= 0.002), hs-CRP (rho= 0.486 p= 0.035) and uremia (rho= 0.476 p= 0.039). After a multiple linear regression test, a positive association was noted between the reactive hyperemia index and the systolic blood pressure (β 0.049, 95% CI [0.011 - 0.087]; p= 0.015).

**Conclusion:**

the endothelial dysfunction is a new concept in the management of rheumatoid arthritis. Screening and detection of this endothelial dysfunction in the subclinical state is possible thanks to new tools including EndoPATH2000. This could make it possible to predict the risk of cardiovascular events notably high blood pressure in general, and high systolic blood pressure in particular.

## Introduction

The vascular endothelium is an anatomical barrier between circulating blood and vascular smooth muscle cells. Furthermore, it plays an essential role in many basic vascular physiological regulations. It is now recognized as a homeostatic organ essential to cardiovascular biomechanics by participating in the structural and functional regulation of vessels [1]. Under normal conditions, blood pressure and blood flow are the major factors influencing endothelial biology either directly through blood flow (mechanical stimuli) or indirectly through chemical factors (chemical stimuli) [2]. Rheumatoid arthritis is the most common chronic inflammatory arthritis in adults [3]. It is generally characterized by high morbidity and mortality with a considerable reduction in life expectancy [4]. Cardiovascular events are the leading cause of increased mortality seen in rheumatoid arthritis [5]. They would be responsible for 30 to 50% of all deaths linked to rheumatoid arthritis [6]. In the prospective NHS (Nurses´ Health Study), patients with rheumatoid arthritis were shown to have a risk of stroke more than twice as high as patients without rheumatoid arthritis [7]. These vascular accidents are generally of atheromatous origin [8]. Recently, studies have shown that atherosclerotic lesions occur earlier and progress more rapidly in patients with rheumatoid arthritis than in the general population [6].

Endothelial dysfunction is considered a key event in the development of atherosclerosis [9]. Authors have reported that atherosclerosis begins with endothelial dysfunction [10,11]. The term endothelial dysfunction describes any form of functional and reversible alteration of endothelial cells [12], resulting in reduced endothelium-dependent vasodilation, a pro-inflammatory state, and proliferative and pro-thrombotic properties [13]. Endothelial dysfunction is an important early event in the pathogenesis of atherosclerosis, contributing to plaque initiation and progression [9]. Thus, endothelial dysfunction could constitute a therapeutic target or better a predictive risk marker of cardiovascular events in patients with rheumatoid arthritis. In addition, the early detection and correction of endothelial dysfunction could be a preventive solution for atherosclerosis and would help reduce cardiovascular risk in rheumatoid arthritis [14].

Authors have reported in the literature a number of data relating to the existence of an association between autoimmune connective tissue diseases and endothelial function. Data suggest that inflammation caused by these diseases can lead to endothelial dysfunction [15], through the action of pro-inflammatory cytokines which can alter the vascular system of nitric oxide release [16] and endothelium derived hyperpolarizing factor [17], causing alteration of dependent endothelium vasodilation. Altered endothelial reactivity has been demonstrated during rheumatoid arthritis but in patients with high disease activity. Most of the time, diagnostic means of endothelial dysfunction are operator dependent in nature as ultrasound measurement of flow-mediated vasodilation, and can only identify clinically manifest endothelial dysfunctions. In the present study, we want to show the possibility and the importance of detecting endothelial dysfunction at the sub-clinical stage even outside the phases of disease activity, and above all with an easy, fast, non-invasive and non-operator dependent way. This study aims to evaluate endothelial function to detect subclinical dysfunction during rheumatoid arthritis in Black African subjects by non-invasive measurement of peripheral arterial tone (vasodilator responses to vascular occlusion) using a device called EndoPAT2000®.

## Methods

### Study design and setting

This is a case-control and prospective study, conducted in the service of human physiology and function testing in faculty of medicine, pharmacy and dentistry in the Cheikh Anta Diop University of Dakar in Senegal. The patients were recruited from rheumatology services in Aristide LeDantec Hospital of Dakar, Senegal. Rheumatoid arthritis is a major cause of often fatal cardiovascular complications. Early detection of these complications would be of crucial importance in the secondary prevention of this condition.

### Study population

The study included 19 subjects aged 18 years and older. We did random sampling. The parameters for this study were reported in a single medical visit. We included subjects with rheumatoid arthritis without clinically detectable cardiovascular complications. Were excluded patients under 18 years and those whose rheumatoid arthritis was associated with severe complications (severe joint deformities in the hands, ischemia, gangrene), or subjects in the activation phase of the disease, or pregnancy/breastfeeding, or other conditions (other primary or secondary connective).

### Data collection

Sociodemographic information and those concerning the history of the disease were collected by questionnaire and medical review. For each patient included, were measured: i) The weight with a Secca® brand personal scale; ii) The hight and the waist size with a rubanmetre. The body mass index (BMI) and heart rate were automatically calculated by the EndoPAT2000®. Systolic blood pressure (SBP) and diastolic blood pressure (DBP) were measured manually with a Spengler® type sphygmomanometer after 10 min of rest. Its were measured before the test with the EndoPAT2000®. Mean blood pressure (MBP) was calculated using the formula of Messaï E. Arnette Ed Blackwell (Paris). 1995: MBP = (SBP+2 DBP)/3.

For the assessment of endothelial function, we used a new device EndoPAT2000® Itamar brand. It is a device for measuring the peripheral arterial tone by vasodilatory response to 5 min occlusion of the brachial artery vascular flow using an inflatable cuff placed on the arm. Reactive hyperemia in response to the occlusion is calculated automatically by the device and translated into reactive hyperemia index (RHI) and its logarithm (LnRHI). The calculated values are normalized by measures on the contralateral arm where the blood flow is not interrupted. The measurement technique was made according to the recommendations of Goor *et al*. [18]. After each measurement, the software installed in a PC and connected to the device, directly expresses the results giving RHI and LnRHI whose normal values are respectively (1.67 to 2) and (0.51 to 0.70). A RHI value less than 1.67 or LnRHI value less than 0.51 may be considered as an endothelial dysfunction. In this work, we consider the RHI.

### Laboratory analysis

The biological parameters were measured the same day in the Laboratory of Biochemistry of Faculty of Medicine, Pharmacy and Dentistry, UCAD of Dakar, Senegal. The takings were made at 8 am in the morning after a fat of at least 12 hours. Some venous blood was taken at the level of the fold of the elbow of the not dominant arm. So on fluoride tube, we measured the fasting blood sugar. Using dry tubes, we measured serum calcium and hsCRP. On heparine tube we measured transaminases (Alanine AminoTransferase: ALT and ASpartate AminoTransférase: AST), Cholesterol Total, HDL-Cholesterol and Triglycerides, serum creatinine and Uremia. By enzymatic method, we measured the fasting blood sugar, the transaminases (ALT and AST), the Total Cholesterol, the HDL-Cholesterol, the LDL-Cholesterol, the Triglycerides, the Urea and the Serum Creatinine. By chemical method, we measured Calcemia. By turbidimetric method, we measured C-reactive Protein. Low-density lipoprotein cholesterol (LDL cholesterol) level was calculated by the Friedewald formula: LDL cholesterol = Total cholesterol - HDL cholesterol - Triglycerides/5.

### Definitions

**Rheumatoid arthritis (RA)** is a chronic inflammatory rheumatism of adults. It is an autoimmune, multifactorial pathology characterized by joint damage and is also accompanied by extra-articular manifestations, notably cardiovascular events. It is considered a cardiovascular risk factor.

**The vascular endothelium** is a layer of epithelial cells that lines the inside of the walls of the heart and blood vessels. It constitutes a real interface between the constituents of the vascular wall and the circulating blood. The endothelium plays a key role in the regulation of vascular tone and the control of hemostasis. It is involved in the control of vasomotor function and blood coagulation.

**The Reactive hyperemia index (RHI) or EndoScore** explores the peripheral arterial tone, a mark of endothelial vasodilator function. It reflects endothelium-dependent vasodilation of systemic blood vessels and accurately reflects the endothelial reactivity. Its normal values are between 1.67 and 2. RHI value below 1.67 are categorized as endothelial dysfunction. It makes it possible to detect subclinical Atherosclerosis.

**Atherosclerosis** is considered a chronic inflammatory disease triggered by damage to the arterial wall endothelium (endothelial dysfunction as the first stage of the atherosclerosis). This endothelial dysfunction causes changes in the permeability of the arterial wall, increased expression of several adhesion molecules on the surface of cells with cytokines production, increased migration of monocytes and T lymphocytes from the circulation towards the arterial wall. This results in a deposition of atherosclerotic plaque, essentially composed of lipids, on the wall of the arteries. Ultimately, these plaques can cause damage to the arterial wall (sclerosis) leading to obstruction of the vessel, or even rupture, with often dramatic consequences.

### Statistical analysis

Analyzes were performed using IBM SPSS Statistics version 25.0.0.0. Qualitative variables are expressed as a percentage. Quantitative variables are expressed as a means and standard deviations. Spearman correlation test, simple and multiple linear regression tests were used to assess the relationships between the reactive hyperemia index (RHI) and the other variables studied. The significance levels were set at p<0.05.

### Ethical considerations

All recruited subjects were informed about the interest of this study, and all signed a consent form. All procedures were conducted in accordance with the standards of the Declaration of Helsinki. It was reviewed and approved by the Ethics Committee of UCAD.

## Results

### General characteristics of the study population

The mean age of the study population was 42.84 ± 12.80 years. The sex ratio (M/F) was 0.19. We find that 81% of patients had a rheumatoid arthritis with a presence of rheumatoid factor. The mean dose of corticosteroid was 10 mg / day. None of the men had a family history of autoimmunity or a history of cardiovascular risk factors. Additionally, none of the men had rheumatoid arthritis with the presence of rhumatoid factor (Table 1). The mean of the parameters was expressed by gender in the Table 2. We found that the BMI mean of the total population was within the normal range. However, if we considered women only, the BMI mean was slightly above normal. At the same time, the waist size circumference mean was also higher for women. The hs-CRP testing showed a higher than normal levels in thirteen patients (13) or 68.42% of the study population. Serum calcium, fasting blood glucose, lipid profile, kidney function and the rest of the laboratory tests were normal in all subjects.

**Table 1:** characteristics of the patients and the rheumatoid arthritis disease

Variables	Women N=16	Men N=3	Total Population N=19
**Background and Cardiovascular risk factors**			
Family Autoimmunity n (%)	4 (25.00%)	-	4 (21.05%)
Hypertension n (%)	3 (18.80%)	-	3 (15.79%)
Dyslipidemia n (%)	2 (12.50%)	-	2 (10.53%)
**Characteristics of rheumatoid arthritis**			
Positive RF (UI/mL) n (%)	13 (81.20%)	-	13 (68.42%)
Positive Ab anti-PC (UI/mL) n (%)	16 (100%)	3 (100%)	19 (100%)
Disease duration (months) Means ± SD	43.00 ± 41.31	24.33 ± 0.58	40.05 ± 38.36
Corticosteroid duration (months) Means ± SD	24.25 ± 36.69	25.00 ± 1.0	24.37 ± 33.50
Corticosteroids dose (mg/day) Means ± SD	9.69 ± 1.25	8.33 ± 2.89	9.47 ± 1.58
Immunosuppressive duration (months) Means ± SD	24.54 ± 41.01	13.50 ± 16.26	23.07 ± 38.42
Immunosuppressive dose (mg/week) Means ± SD	14.23 ± 0.53	15.00 ± 0.00	14.33 ± 0.45

RF=Rhumatoid Factor, Ab anti-PC= Antibodies anti-proteines citrullinated, SD=Standard Deviation

**Table 2:** frequency and means of anthropometric and clinical characteristics of the study population

Variables	Women (N=16) Means ± SD	Men (N=3) Means ± SD	Total pop (N=19) Means ± SD
**Anthropometric characteristics**			
Body mass index (Kg/m^2^)	25.04 ± 1.22	20.11 ± 2.19	24.26 ± 1.14
Waist size (cm)	81.00 ± 12.75	75.67 ± 7.02	80.16 ± 2.76
Hip circumference (cm)	102.06 ± 10.19	94.00 ± 5.57	100.79 ± 2.28
**Cardiovascular parameters**			
Heart rate (bpm)	82.00 ± 13.43	78.67 ± 21.39	81.47 ± 3.27
Systolic blood pressure (mm Hg)	134.75 ± 25.93	133.00 ± 9.85	134.47 ± 5.49
Diastolic blood pressure (mm Hg)	82.13 ± 13.01	79.00 ± 4.00	81.63 ± 2.76
Mean blood pressure (mm Hg)	99.60 ± 16.27	97.00 ± 5.90	99.25 ± 3.44
Reactive hyperhemic index	1.58 ± 0.87	1.66 ± 0.69	1.57 ± 0.83
**Biochemical parameters**			
Calcemia (mg/l)	85.14 ± 2.49	86.77 ± 1.90	85.31 ± 8.81
Fasting blood glucose (g/l)	0.93 ± 0.09	0.69 ± 0.04	0.91 ± 0.12
Uremia (g/l)	0.21 ± 0.06	0.21 ± 0.12	0.21 ± 0.06
Creatinine (mg/l)	6.66 ± 1.31	6.61 ± 2.08	7.24 ± 1.69
Total cholesterol (g/l)	2.20 ± 0.24	1.89 ± 0.16	2.07 ± 0.31
HDL cholesterol (g/l)	0.64 ± 0.12	0.59 ± 0.04	0.58 ± 0.13
LDL cholesterol (g/l)	1.25 ± 0.23	1.10 ± 0.12	1.34 ± 0.30
Triglycerides (g/l)	0.79 ± 0.30	0.68 ± 0.18	0.76 ± 0.29
High sensitive C-reactive protein (mg/l)	14.26 ± 14.75	27.76 ± 15.74	12.48 ± 14.22
Aspartate amino-transferase (g/l)	15.18 ± 2.38	19.97 ± 10.69	19.40 ± 9.02
Alanine amino-transferase (g/l)	14.01 ± 3.31	19.17 ± 11.47	16.06 ± 8.37

### Prevalence and correlates of Endothelial dysfunction

More than half of subjects, 57.89% of the study population, had an RHI value below the normal value. They had an impaired endothelial function (Table 2). Thanks to a Spearman correlation test, considering the entire study population, positive correlations were found between RHI and systolic blood pressure, mean blood pressure, hsCRP and urea (Table 3). After multiple linear regression test, only systolic blood pressure remains positively associated with reactive hyperemia index and the systolic blood pressure (β 0.049, 95% CI [0.011 - 0.087]; p = 0.015), as shown in Table 4.

**Table 3:** factors involved in impaired endothelial function

Reactive hyperhemic index		
**Variables**	**Correlation coefficient**	**p-value**
Age (years)	rho = - 0.072	0.768
Body mass index (kg/m^2^)	rho = 0.055	0.822
Waist size (cm)	rho = 0.033	0.893
Heart rate (bpm)	rho = - 0.246	0.309
Systolic blood pressure (mm Hg)	**rho = 0.753**	0.0002*
Diastolic blood pressure (mm Hg)	rho = 0.385	0.103
Mean blood pressure (mm Hg)	**rho = 0.660**	0.002*
Fasting blood glucose (g/l)	rho = 0.029	0.906
Serum creatinine (mg/l)	rho = - 0.169	0.488
Uremia (g/l)	**rho = 0.476**	0.039*
Total cholesterol (g/l)	rho = 0.023	0.926
HDL cholesterol (g/l)	rho = - 0.136	0.579
LDL cholesterol (g/l)	rho = - 0.035	0.888
Triglycerides (g/l)	rho = - 0.080	0.745
High-sensitive C-Reactive Protein (g/l)	**rho = 0.486**	0.035*

* statistically significant difference

**Table 4:** both simple and multiple regression analysis for RHI

Variables	RHI related			
	Unadjusted ORs (95% CI)	p-value	Adjusted ORs (95% CI)	p-value
PAS (mm Hg)	0.026 [0.013 - 0.038]	0.0003*	0.049 [0.011 - 0.087]	0.015*
MBP (mm Hg)	0.034 [0.011 - 0.056]	0.006*	-0.045 [-0.107 - 0.017]	0.142
hsCRP (g/l)	0.013 [-0.014 - 0.040]	0.311	-0.001 [-0.022 - 0.020]	0.939
Urea (g/l)	5.462 [-0.385 - 11.308]	0.065	2.543 [-2.860 - 7.946]	0.330

**PAS:** systolic blood pressure; **MBP**: mean blood pressure; **hsCRP:** high sensitive C-Reactive Protein; *statistically significant difference

## Discussion

Endothelial dysfunction would be the bedrock of atherosclerosis which would constitute an independent factor of cardiovascular events during rheumatoid arthritis. The post-occlusion reactive hyperemia index (RHI) is a reflection of endothelium-dependent vasodilation. A decrease in the RHI below 1.67 indicates endothelial dysfunction. Our study shows that the post-occlusion reactive hyperemic index was abnormally reduced in 57.89% of all subjects with rheumatoid arthritis. This means that more than half of polyarthritic subjects had an endothelial dysfunction. Thus, these results may suggest impaired endothelium-dependent arterial vasodilation in rheumatoid arthritis. We found a positive correlation between reactive hyperemia index with systolic blood pressure (rho=0.753 p=0.0002), mean blood pressure (rho=0.660 p=0.002), high sensitive c-reactive protein (rho=0.486 p=0.035) and the uremia (rho=0.476 p=0.039). These results are consistent with the literature. Endothelial dysfunction in rheumatoid arthritis has been described for the first time in 2002 by Bergholm *et al*. [19], who reported a decrease in the acetylcholine-dependent vasodilation in patients with rheumatoid arthritis compared to control subjects. This result was confirmed by other studies such as those which had shown the possibility of a decrease in endothelium-dependent vasodilation [20]. However, the results of studies on endothelial function in rheumatoid arthritis are controversial. Other studies have noted no change in endothelial function in rheumatoid arthritis [21]. It is well established that the endothelium is capable of synthesizing and releasing different mediators. They are likely to have effects on vascular and circulating cells and affect vascular function as vasomotricity [22]. These mediators are either vasoconstrictor (endothelin) or vasodilator (nitric oxide: NO). Indeed, NO is an important vasoactive factor that regulates the vasodilating action of endothelial cells on smooth muscle cells but also inhibits platelet aggregation and leukocyte adhesion to vascular wall [2]. Endothelial dysfunction indicates a secretory abnormality of one or more of these mediators. In parallel, the alteration of endothelium-dependent vasodilation is generally the prerogative of an anomaly in the bioavailability of endothelial nitric oxide (NO) [5].

However, there are other biomarkers of endothelial function (Vascular Cell Adhesion Molecule [VCAM], Inter Cellular Adhesion Molecule [ICAM-1], Asymmetric dimethylarginine [ADMA], Von Willebrand factor, circulating endothelial cells) which deserve to be studied. to better elucidate this endothelial dysfunction in rheumatoid arthritis [2]. ADMA is a potent endogenous nitric oxide synthase (NOS) inhibitor in the blood, increased plasma levels reflect endothelial dysfunction. It has been evaluated in rheumatoid arthritis and it seems to be a predictor of the development of endothelial dysfunction in recent rheumatoid arthritis without disease or cardiovascular risk factors [23]. It turns out that RHI measurement could detect endothelial dysfunction with the same precocity and in a faster, more reliable and cheaper way. The increase in these adhesion molecules was observed as well as a strong correlation of plasma concentrations with markers of inflammation in rheumatoid arthritis compared with a group of healthy control subjects. Meanwhile, other authors suggest the possibility of complex interactions between environmental and genetic determinants affect both the immune system and the vascular system to modify the cardiovascular risk in rheumatoid arthritis [2]. Authors have suggested the existence of several factors favoring endothelial dysfunction during rheumatoid arthritis such as systemic autoimmune processes, in which autoantibodies can play a direct role in endothelial damage [24]. Similarly, Sandoo *et al*. recently demonstrated a link between endothelial dysfunction and active progression of rheumatoid arthritis [25]. In addition, traditional risk factors such as chronic systemic inflammation could be incriminated in endothelial dysfunction in rheumatoid arthritis. It is considered to be a phenomenon that would induce pro-atherogenic changes including endothelial dysfunction [26]. Furthermore, Prati *et al*. conclude that endothelial dysfunction appears to worsen with disease duration [2].

However, these results are limited by the absence of a control group, the small sample sizes and the lack of evidence on the integrity of smooth muscle cells. As a strength of this study, the role of endothelial dysfunction has been strongly suggested to explain the development of vascular complications in rheumatoid arthritis as shown in the study by Bacon *et al*. [27]. In this study, the diagnosis of endothelial dysfunction in the subclinical state was carried out, in a fast, easy, non-invasive and non-operator dependent way, thanks to a new device called EndoPAT2000®. This new method could be an early clinical screening tool for atherosclerotic vascular dysfunction in rheumatoid arthritis. This study would be one of the first to describe endothelial dysfunction in rheumatoid arthritis using an EndoPAT2000®. It also showed the possibility of screening for atherosclerosis at the sub-clinical stage during rheumatoid arthritis. It therefore has a diagnostic and predictive value of cardiovascular events in patients followed for rheumatoid arthritis. The strengths of this study lie in the fact that it is the first to study endothelial dysfunction during rheumatoid arthritis in a Senegalese population. It also allowed the screening of patients in the subclinical state at the start of cardiovascular complications.

## Conclusion

Endothelial dysfunction is a new concept in rheumatoid arthritis. Its highlighting allows to predict the cardiovascular risk and to assess the functional vascular damage degree. Our study shows that screening and detection of endothelial dysfunction at a subclinical state is possible using new tools including EndoPATH2000®. This endothelial dysfunction is associated with high blood pressure in general and high systolic blood pressure in particular. The assessment of endothelial dysfunction by measuring the endothelium-dependent vasodilatory response to occlusion may therefore be one of the non-invasive and physical methods that are recent and innovative.

### What is known about this topic

- Polyarthritis is a chronic inflammatory disease causing cardiovascular complications;
- The initial stage of vascular damage is endothelial dysfunction.

### What this study adds

- Non-invasive, rapid and non-operator-dependent assessment of endothelial dysfunction;
- Early detection of endothelial dysfunction in the subclinical state.
